# A brain-computer interface driven by imagining different force loads on a single hand: an online feasibility study

**DOI:** 10.1186/s12984-017-0307-1

**Published:** 2017-09-11

**Authors:** Kun Wang, Zhongpeng Wang, Yi Guo, Feng He, Hongzhi Qi, Minpeng Xu, Dong Ming

**Affiliations:** 1 Lab of Neural Engineering & Rehabilitation, Department of Biomedical Engineering, College of Precision Instruments and Optoelectronics Engineering, Tianjin University, Tianjin, China; 2 Tianjin International Joint Research Center for Neural Engineering, Academy of Medical Engineering and Translational Medicine, Tianjin University, Tianjin, China

**Keywords:** Force load, Motor imagery, Electroencephalogram (EEG), Event-related Desynchronization (ERD), Brain-computer Interface (BCI)

## Abstract

**Background:**

Motor imagery (MI) induced EEG patterns are widely used as control signals for brain-computer interfaces (BCIs). Kinetic and kinematic factors have been proved to be able to change EEG patterns during motor execution and motor imagery. However, to our knowledge, there is still no literature reporting an effective online MI-BCI using kinetic factor regulated EEG oscillations. This study proposed a novel MI-BCI paradigm in which users can online output multiple commands by imagining clenching their right hand with different force loads.

**Methods:**

Eleven subjects participated in this study. During the experiment, they were asked to imagine clenching their right hands with two different force loads (30% maximum voluntary contraction (MVC) and 10% MVC). Multi-Common spatial patterns (Multi-CSPs) and support vector machines (SVMs) were used to build the classifier for recognizing three commands corresponding to high load MI, low load MI and relaxed status respectively. EMG were monitored to avoid voluntary muscle activities during the BCI operation. The event-related spectral perturbation (ERSP) method was used to analyse EEG variation during multiple load MI tasks.

**Results:**

All subjects were able to drive BCI systems using motor imagery of different force loads in online experiments. We achieved an average online accuracy of 70.9%, with the highest accuracy of 83.3%, which was much higher than the chance level (33%). The event-related desynchronization (ERD) phenomenon during high load tasks was significantly higher than it was during low load tasks both in terms of intensity at electrode positions C3 (*p* < 0.05) and spatial distribution.

**Conclusions:**

This paper demonstrated the feasibility of the proposed MI-BCI paradigm based on multi-force loads on the same limb through online studies. This paradigm could not only enlarge the command set of MI-BCI, but also provide a promising approach to rehabilitate patients with motor disabilities.

## Background

In the past several decades, an increasing number of researchers have focused on decoding information from brain which could be applied to construct brain-computer interfaces (BCIs). BCIs can provide a direct communication pathway between the brain and external devices without using peripheral nerves and muscles [1]. Motor imagery-based BCI (MI-BCI) is one of the most important BCI paradigms. An outstanding advantage of the paradigm is that it requires no real action from users. It has been demonstrated that motor imagery could induce the event-related de-synchronization/synchronization (ERD/ERS) phenomena occuring at 8-13 Hz (mu rhythm) and 14-30 Hz (beta rhythm) [2], which could be reliably recognized through appropriate algorithms, such as, power spectral density, source imaging method and so on.

Several clinical applications of MI-BCI systems have been reported. MI-BCI not only can be used as a communication or control methods to help patients with serious movement disorder like ALS [3], cerebral palsy [4], etc. to complete the daily interactions. More importantly, MI-BCI is more and more used in the recovery of stroke. Several studies have confirmed that MI-BCI is an effective method for post stroke rehabilitation [5–7]. In these studies, a variety of functional devices, such as functional electrical stimulation (FES) [8], rehabilitation robots [9], etc., were used in combination with MI-BCI to construct a close loop neurofeedback from the sensorimotor cortex to paralyzed limbs [8–11].

Novel motor imagery paradigms have been designed to decode motion intention accurately and to improve MI-BCI control performance efficiently. Compound and sequential limb motor imagery have been proved to be divisible as well as the simple limb [12, 13]. The combination of simple and compound limb motor imagery enabled the MI-BCI to control a quadcopter with three-dimensional movements [14]. In recent years, many researchers made attempt to decode the fine motion intentions on the single limb, aiming at building the connection between the MI task and the corresponding action of the output device. Edelman et al. have classified four different postures MI tasks of the right hand with a high accuracy using an EEG source imaging method [15]. Many researchers have demonstrated that the EEG pattern could reflect the kinematic and kinetic features of movements. For example, a linear correlation between ERD and the speed of hand grasping movements was found both during actual execution and motor imagery [16]. Nakayashiki et al. [17] showed that the time differentiation in kinematics (change of hand posture) was related to mu/beta-ERD strength. Movement-related cortical potential (MRCP) was used to detect the motion intentions associated with different levels of speed and force of the right ankle [18]. These studies implied the feasibility of multiple BCI commands based on kinematic or kinetic factors of motor imagery on the same limb. Due to the characteristics of hemiplegia for stroke patients as well as the attention for the kinematic and kinetic indicators in rehabilitation, it has potential value for the MI-BCI decoding of motion factors. Compared to other methods, MI-BCI could assist post stroke patients with hemiplegia by enhancing the motor imagery of the affected limb and could significantly improve the recovery of the damaged sensorimotor cortex.

However, at present, there is a scarcity of reported online studies, which verify the feasibility of the paradigm with motion factor regulated MI on a single limb. Moreover, a number of inconsistent previous results lead to uncertain conclusions.

For example, EEG and fMRI studies showed that a significant higher activation occurs under a higher force load [19, 20]. However according to certain studies no significant difference was observed in the different motor loads under force hold conditions [17]. Although the experimental conditions and analytical methods vary among these studies, further discussion to ensure the feasibility of motion factor related MI-BCI is necessary.

In this paper, we investigated one kinetic factor, i.e. the force load regulated during motor imagery of one limb. Unlike several previous studies, we used the online BCI experiment to prove the feasibility of multi-force BCI based on motor imagery.

## Methods

### Participants

Eleven subjects (2 females and 9 males, 21 ~ 25 years old) participated in this experiment. All subjects are healthy without any history of neurological illness or limb movement disorders. They were all informed about the procedure of the study and were required to undergo training before the formal experiment. They were all informed about the procedure of the study and were required to have training before the formal experiment. Each subject would be proficient in imagining different force intensities after about an hour training of alternatively clenching and imaging their right hands. All subjects completed the movement imagery questionnaire and provided written consent before the experiment began.

### Experimental procedure

During the experiment, the subjects were required to sit in a comfortable chair straight up with the forearm flexed (90°) to horizontal supported by a bracket. Both the forearm and the wrist were in the neutral position. An electrode cap was placed on their head and two electrodes posted on the extensor carpi radialis longus of their right hand to confirm the clear presence of muscle contraction [21]. A computer screen was placed in front of them at a distance of about 1 m to display visual cues. Before the experiment commenced, the subjects were asked to measure the maximum voluntary contraction (MVC) of their right hand. An electrical dynamometer was used to measure and display the values of the clenching force. Kilogram-force (kgf) used as the unit of force. Subjects performed isometric muscle activations (activation without limb movement) of the hand and wrist. We chose 30% MVC (12 ~ 15 kgf for males and 6.6 ~ 7.8 kgf for females) as the high clenching force load, at which the subjects were able to exert sufficient effort while being capable of completing the task without inducing any fatigue, while 10% MVC (4 ~ 5 kgf for males and 2.2 ~ 2.6 kgf for females) was selected as the low clenching force load, at which the subjects were able to exert medium effort to complete the task. Hence, motor execution tasks during the experiment consisted of three stages: high load, low load and relaxed states, corresponding to 30% MVC, 10% MVC and 0% MVC respectively. Furthermore, the subjects were also required to carry out two motor imagery tasks during this experiment. They were required to imagine clenching their hand with the two force load levels which were similar to the motor execution tasks. During the motor execution task, the subjects were required to watch the dynamometer displayer, burst up to the required level and maintain their clenching force.

**Fig. 1:**
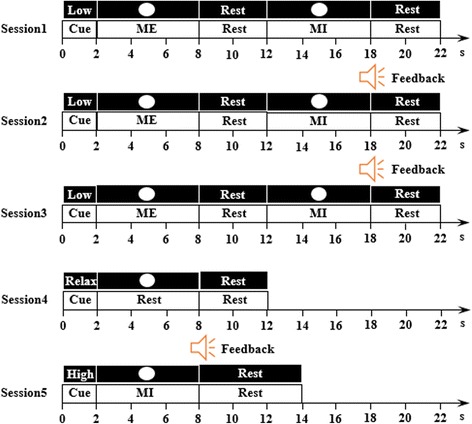
The timeline of one trial of the experimental paradigm. Sessions 1, 2 and 3 have two tasks, high force load and low force load. Each trial has a motor execution task followed by a motor imagery task at the same force load. There were two blocks during these sessions and each block included 10 trials (5 trials of high and 5 trials of low, randomly sorted). Session 4 has only one task, relaxed (30 trials). Session 5 has three tasks (high, low and relaxed). It consisted of four blocks, each of which included 12 trials (4 trials for high, 4 trials for low and 4 trials for relaxed, randomly sorted). Sessions 2, 3 and 5 present a voice feedback after each motor imagery task to indicate whether the classifier correctly identified the imagery force load level

The experiment consisted of 5 sessions as shown in Fig. 1, Sessions 1, 2 and 3 involved two types of tasks during one trial, i.e. motor execution and motor imagery. Each trial began with a word cue on the screen for 2 s to indicate whether its force load was high or low. Next, a white circle appeared at the center of the screen for 6 s. During this period, the subjects were instructed in motor execution, i.e. clenching their hand with the required level of force. Then, a 4 s ‘Rest’ period followed, during which the subjects relaxed. A white circle then appeared again for 6 s and the subjects were required to imagine clenching their hand with the same force load as the last motor execution period but to avoid any real motion. At the end of the trial a 4 s ‘Rest’ period followed during which the subjects relaxed again. There were two blocks during these sessions and each block included 10 trials (5 trials of high and 5 trials of low, randomly sorted).

The purpose of this paradigm design involving motor execution followed by motor imagery with the same load during one trial was to attempt to ensure the subjects were able to imagine ‘correctly’ [22]. Furthermore, to enhance the specificity of the motor imagery, feedback was added to sessions 2 and 3 of the experiment. A two-class classifier, high-versus-low, was built using the data from session 1, following which the motor imagery was classified by it. During sessions 2 and 3, voice feedback containing the classification result was presented to the subjects at the end of each trial to notify them of whether their motor imagery had been correctly identified. The subjects could adjust their imagination following the feedback during the next trial and were required to achieve the highest possible classification accuracy.

Session 4 consisted of 30 trials for the resting condition (‘relaxed’ task). This session involved the collection of relaxed status data, which was used to build a three-class classifier, i.e. high, low and relaxed, combined with the data from the motor imagery of sessions 1 to 3. Session 5 involved the online experiment to evaluate the three-class classification. It consisted of four blocks, each of which included 12 trials (4 trials for high, 4 trials for low and 4 trials for relaxed, randomly sorted). Compared with the two-class experimental stage, there were no motor execution tasks during each trial. Subjects were required to imagine three tasks, i.e. high load, low load and relaxed, without the motor execution referenced during each trial. Voice feedback was presented following motor imagery during each trial.

Throughout the rest of this paper, MEH (motor execution high), MEL (motor execution low), MIH (motor imagery high), MIL (motor imagery low) and RX (relaxed) are used to depict the five tasks respectively. As described above, the motor imagery tasks occurred in two stages, sessions 1 to 3 served as the first stage, during which MI tasks followed ME tasks, while session 5 served as the second stage, during which MI tasks operated independently. Correspondingly, we use MIH-1 and MIL-1 to depict MI tasks during sessions 1 to 3 and use MIH-2 and MIL-2 depict MI tasks during session 5.

### Signal recording

EEG signals were recorded from 64 Ag/AgCl scalp electrodes placed according to the international 10-20 system. The reference electrode was placed at the nose and the ground electrode was placed at the prefrontal lobe. Raw EEG signals were acquired at a sampling frequency of 1000 Hz with a SynAmps2 amplifier (NeuroScan 64). At the same time, a 50 Hz notch filter was used to remove power frequency interference. Meanwhile, one-channel electromyogram (EMG) signals were recorded on the extensor carpi radialis longus of the right hand using the same amplifier.

### Signal processing

#### EEG signal processing

We selected 60 channel signals (except M1, M2, HEO and VEO) for analysis. The EEG signals were first down-sampled from 1000 Hz to 200 Hz and then filtered by an 8-30 Hz Butterworth band-pass filter. The common average reference (CAR) was used to spatially filter the all 60 channel EEG signals as follows:

1 $$ {V}_i^{CAR}={V}_i-\frac{1}{n}\sum_{j=1}^n{V}_i $$

Here, *n* represents the number of EEG electrodes, and *V*_*i*_ represents the amplitude of the raw EEG signal on the *i*th electrode. *V*_*i*_^*CAR*^ represents the re-referenced EEG signals.

The event-related spectral perturbation (ERSP) method was used to analyze EEG features on the time-frequency domain, which could present ERD/ERS patterns of different types of motor imagery. ERSP was defined as

2 $$ ERSP\left(f,t\right)=\frac{1}{n}\sum_{k=1}^n\left({F}_k{\left(f,t\right)}^2\right) $$

Here, *n* represents the number of trials, and *F*_*k*_(*f*, *t*) represents the spectral estimation of the *k*th trial at frequency *f* and time *t* [23]. Short-time Fourier transform (STFT) with a 256-point-long Hanning-tapered window was employed to perform the spectral estimation in EEGLAB. To produce the baseline-normalized ERSP values (dB), the mean power changes in a baseline period (2 s before motor imagery onset) were subtracted from each spectral estimation. Mean ERSP values were calculated between frequency ranges of 8 Hz to 30 Hz and time ranges of -2000 ms to 6000 ms for every motor imagery task. ‘0’ referred to the moment when the white circle appeared prompting the subjects began to imagine. In this paper, we used the mean ERSP values of all subjects from electrode C3 to compare the time-frequency variation among the motor imagery for three force loads.

The common spatial pattern (CSP) algorithm [24] was used to maximize the variance of the EEG features among different motor imagery tasks. Two-class CSP was one of the most classic EEG feature extraction methods in motor imagery based BCI. For the 3 tasks in session 5, we used the multiclass CSP method to extract the features [12, 24]. A linear support vector machine (SVM) was used to build the classifier with the help of the famous software package LIBSVM [25]. All programs were compiled and run on the MATLAB platform.

During the experiment, two classifiers were built. The first one was built after session 1 finished. Using EEG data from session 1, a two-class CSP filter was built and a two-class linear SVM was trained. After session 2, the CSP filter and the classifier were updated with the new data. The second classifier was built after session 4, while a three-class CSP filter and a three-class linear SVM were built using EEG data from sessions 1 to 4.

#### EMG signal processing

EMG signals were first processed by 50 Hz notch and filtered by a 5-500 Hz band-pass filter. We calculated the mean integrated EMG (IEMG) as the indicator of the muscle activity during each task. IEMG is capable of reflecting the contraction property of muscles [26]. It is defined as:

3 $$ IEMG=\sum_{i=1}^n\left|{x}_i\right| $$

Here, *x*_*i*_ represents the *i*th sample of EMG signal and *n* represents the length of one trial.

## Results

### EMG analysis during experimental tasks

**Fig. 2:**
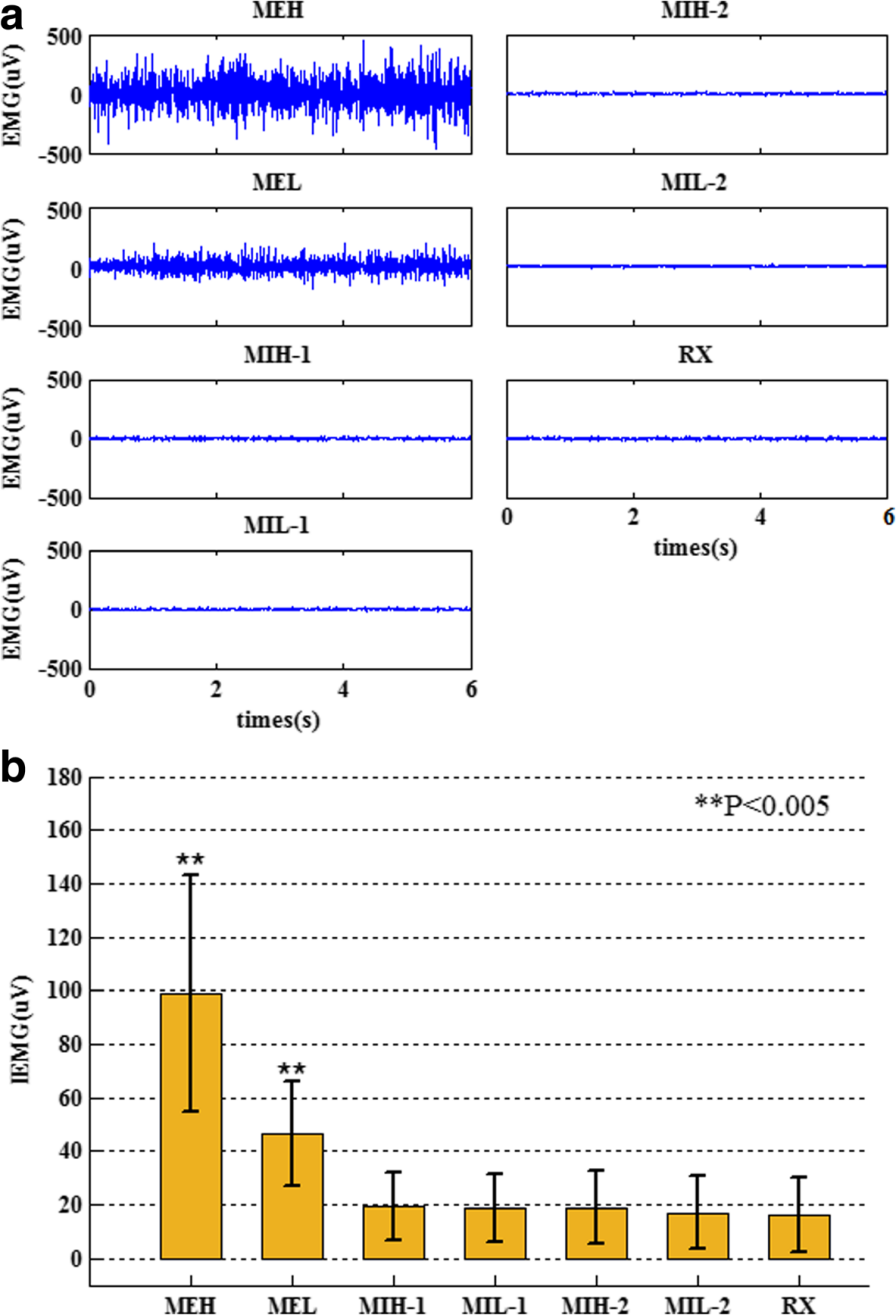
Examples of the EMG signals for one representative subject and the comparison of IEMG values. **a** The EMG signals of one subject during seven experimental tasks. **b** The comparison of IEMG values between RX task and other tasks. MEH and MEL show significant differences compared to relaxed tasks, while other tasks, motor imagery tasks, show minimal differences

During the experiment, we recorded the EMG signals of each subject on the right hand to ensure muscle activity was avoided under motor imagery conditions. Figure 2a shows the EMG signals acquired from one subject during seven tasks: motor execution tasks present a significantly higher amplitude than other tasks. Figure 2b shows the mean IEMG and the comparison between motor tasks and relaxed tasks. From Fig. 2b, we can observe that there are significant differences between ME tasks (*p* < 0.005, Paired T-test), while MI tasks show no significant differences (*p* > 0.05, Paired T-test). This result shows that the subjects who participated in our experiment successfully avoided muscle contractions during MI tasks.

### Classification performance

**Fig. 3:**
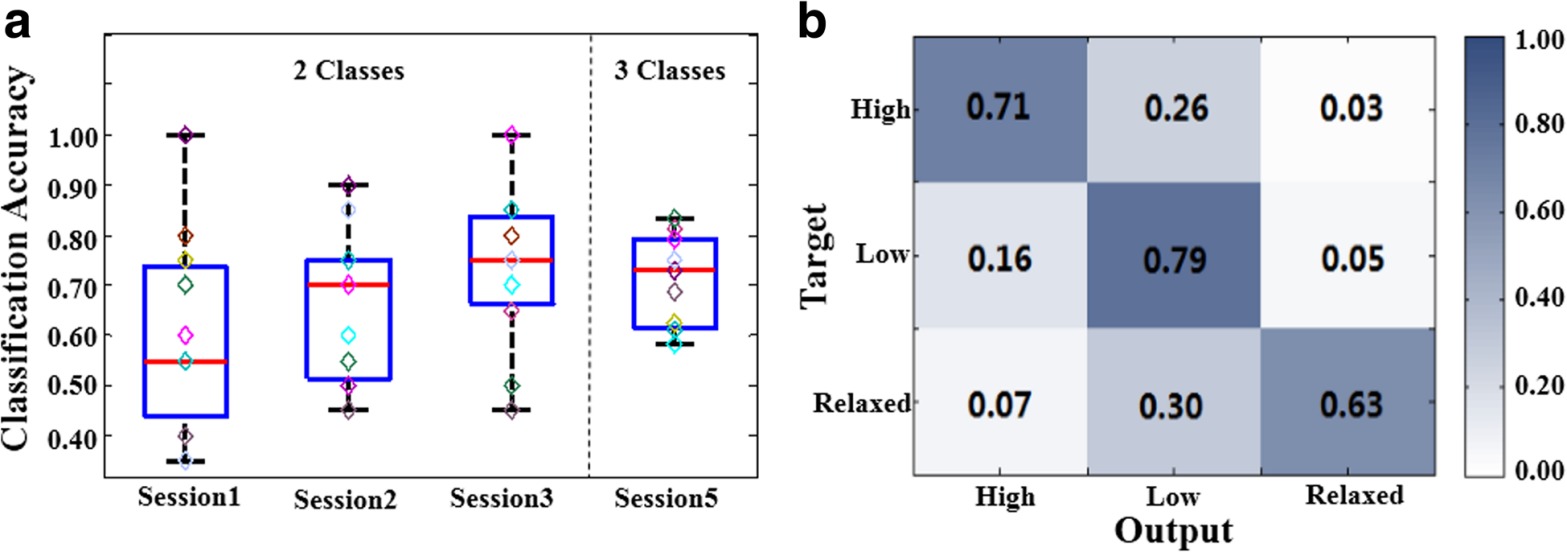
Classification performance. **a** Classification accuracies for each subject during the experiment (each color represented one subject). Session 1 to 3 identify two classifications, ‘high’ versus ‘low’, while session 5 identifies three classifications, ‘high’ versus ‘low’ versus ‘relaxed’. **b** Online output distribution in session 5

Figure 3a shows the classification accuracy in four sessions of the experiment. The accuracy of session 1 is an offline result, while the other sessions are online results with feedback indications. Session 1 to 3 identify two classifications, high-load versus low-load, while session 5 identifies three classifications, high-load versus low-load versus relaxed. The mean classification accuracies are 60.5%, 65.9%, 73.2% during sessions 1, 2 and 3 respectively, during which MI tasks followed ME tasks. During session 5, during which solely MI tasks were carried out, the mean accuracy is 70.9%, with a maximum value of 83.3% and a minimum value of 58.3%. All subjects achieved a significant higher accuracy than the chance selection, 33.3% for three-class identifications during session 5. These results verify that most of the subjects could drive the MI-BCI with an acceptable accuracy using motor imagery of different levels of clenching force loads.

Another phenomenon in Fig. 3a is that the average classification accuracy increases gradually from session 1 to session 3. However the result of repeated-measures ANOVA (*p* = 0.106) and Bonferroni-corrected multiple comparison test (session1 vs session 2 (*p* = 1), session1 vs session 3 (*p* = 0.188), and session 2 vs session 3 (*p* = 0.361)) show there are no significant differences between sessions. But, the paired T-test shows the accuracy in session 3 is higher than in session 1 (*p* = 0.03). During the experiment, these three sessions consisting of the training stage, especially sessions 2 and 3 have the online feedback. The increased accuracy shows that subject gradually became more skilled in controlling their motor imagery or in matching their imagination to classification models.

Figure 3b shows the output distribution of each subject in session 5, which could reflect the inner-category classification results for the three BCI tasks. It shows that the misclassification which occurs among three tasks is imbalanced. For example, when subjects want to output ‘high’, the false output of ‘low’ has a higher occurrence rate (26%) than that of ‘relaxed’ (3%) (Paired T-test, *p* < 0.01). Similarly, when subjects want to output ‘relaxed’, the occurrence rate of false output of ‘low’ is much higher than that of ‘high’ (Paired T-test, *p* < 0.003). Moreover, under the condition of imaging ‘low’, the false output of ‘high’ occurs more frequently than ‘relaxed’, 16% versus 5% (Paired T-test, *p* < 0.03). These results depict that, these three tasks were not scattered in the classifier. More likely, they were located in the same direction, with the ‘high’ farther away from the ‘relaxed’ than the ‘low’.

### Time-frequency analysis of EEG for different mental tasks

**Fig. 4:**
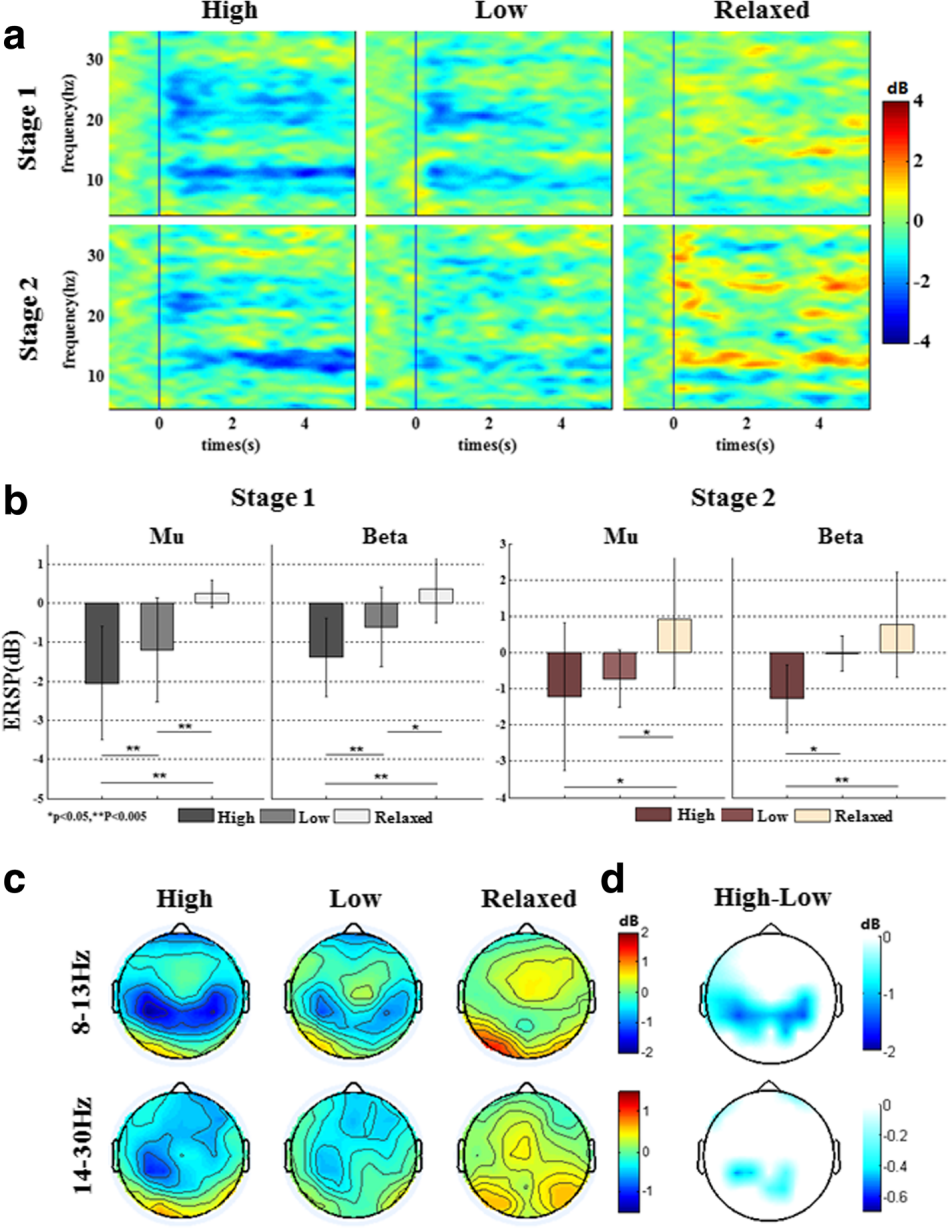
Time-frequency analysis of EEG for different mental tasks. **a** The averaged time-frequency maps on C3 under multiple force load motor imagery tasks. The vertical line was marked at the onset of task, the blue color indicates the ERD phenomenon. **b** The mean ERSP on C3 at mu and beta rhythms during two stages of the experiment. **c** The averaged ERSP topography under multiple force load motor imagery tasks. **d** The significant difference topography between the high force load MI and low force load MI

Figure 4a depicts the averaged time-frequency maps on C3 of all subjects under multi-force load motor imagery tasks in the first stage (session 1 to 3) and second stage (session 4). In this figure, obvious mu and beta ERD (blue indicated ERD) is observed right after the cue (0 s) onset for ‘high’ and ‘low’ force loads during both the first and the second stages. There is a clearer long-lasting power decrease under ‘high’ mental tasks than ‘low’ mental tasks, especially at upper mu rhythm (10-13 Hz). Compared with the ‘low’ task, the ERD phenomenon occurs in broader bands during the ‘high’ task. These results imply the MI patterns of subjects should be correct.

The mean ERSP on C3 of all subjects for the MI and relaxed tasks are shown in Fig. 4b. With the increase in force load for motor imagery tasks, the ERD becomes clearer for all subjects, which reflects a stronger activation of the target motor area for the right hand. It is consistent with the result shown in Fig. 4a. The ERSP reduction on the ‘high’ force load MI is significantly higher (*p* < 0.005) than that for the ‘low’ force load MI at both mu and beta rhythms in the first stage of the experiment. Similarly, at the second stage, the ERSP in the ‘high’ load MI is significantly lower (*p* < 0.05) than that in the ‘low’ load MI in the beta band. To further examine the EEG pattern variance, we calculated the averaged spectral power of each channel in the mu and beta rhythms and compared the results among the MI tasks. Figure 4c shows the mean EEG power topography under different mental force load conditions. We observe that stronger ERD is distributed on the sensorimotor area in the high load MI. Figure 4d shows the difference between ‘high’ load tasks and ‘low’ load tasks. Only the significant differences (Paired T-test, p < 0.05) are kept in this figure. From Fig. 4d, the ERD beta band difference mainly occurs on the left sensorimotor area, however, the mu rhythm ERD difference occurs on both sides of the sensorimotor area. These results show the EEG pattern changes in motor imagery with different force loads and explain the online classification.

## Discussion

In this paper, subjects were trained to imagine clenching their right hand with different force loads. The experimental results showed the EEG patterns significantly changed during different force load motor imagery, and then, with this EEG variance, subjects could drive an online multiple-commands MI-BCI system. Considering commands in traditional MI-BCI always correspond to separate limbs, this study provides a means of extending MI-BCI commands. It is important of MI execution accuracy in experiments. In general terms, subjects would be trained for a period of time before the experiment and researches would evaluate the accuracy of MI execution by the results (such as, if there is the ERD phenomenon, classification accuracy) [14, 27]. In our research, we secured the accuracy of MI execution from training before the experiment, paradigm design (MI after ME) and online feedback.

What’s more, EMG signals were recorded from the right hand in order to assess the absence of muscle tension during MI ensuring the purity of the data.

Although certain previous studies reported that the kinetic and kinematic movement could induce change in EEG patterns or cortical activation, no consistent conclusion has been obtained to date. For example, Pistohl et al. showed a grasping force did not change the ECoG amplitude during reach-to-grasp tasks [28]; Chakarov et al. suggested that EEG spectral power was not affected by different force loads [29]; Nakayashiki et al. also reported that mu and beta band ERDs did not show significant differences between kinetic hand movements [17]. However, Cramer et al. using an fMRI study proved that motor cortex activation varied with three levels of force [19]. Gwin et al. showed that the ERD on both mu and beta rhythms significantly change depending on muscle contraction loads [20]. Jochumsen et al. also reported that muscle contraction with different force loads changes the movement-related cortical potential (MRCP) [18]. These appearances of the kinetic factor may be dependent on several factors, such as experimental paradigms, frequency range, brain regions and averaging protocol. Although the online experiment in this study cannot clarify the controversy, it demonstrates the feasibility of a novel BCI paradigm based on kinetic factors.

In this study, the subjects involved were experienced in MI-BCI but had never participated in multi-force loads experiments. Subjects were trained before the experiment to familiarize themselves with the multi-force loads output. The sessions during the online experiment may also be regarded as a training, during which subjects were able to regulate their imagination in the next trial following indication of voice feedback of the last trial. Previous studies indicated that training did affect cortical activation patterns no matter for the unimpaired subjects [30, 31], or for the stroke patients [5]. Online feedback should also be regarded as another influential factor for the result in this study. Reports showed a significant larger ERD and an increase in specific cortical activation during feedback presentation in motor imagery tasks [32, 33].

As we all know, EEGs have individual differences, especially for the MI tasks. Compared with healthy subjects, stroke patients’ EEGs would be more different due to the diversity of lesion side, hemiplegic limb, stroke onset, age and so on. Although EEG power in the impaired hemisphere was found much lower than that of the healthy side [34], recent studies have revealed that MI related EEG from hemiplegic patients could be used in BCIs [35, 36]. What’s more, MI-BCI application in post stroke rehabilitation has recently become increasingly popular and been confirmed as an effective method of rehabilitation [7, 10, 11]. Nevertheless, ensuring patients engage in MI training with significant effort still remains a problem. ERD refers to the decrease in synchrony of the underlying neuronal populations, which reflect the activation of the cortex [2]. Certain studies attempt to improve the participants’ efforts to induce higher ERD. For example, Prasad el al. designed a computer game-based motor imagery as neurofeedback for post stroke patients to improve their engagement [7]. ERD also could be enhanced using MI-BCI with proprioceptive feedback [37] or haptic feedback by closing the sensorimotor loop [38]. Mostly, MI-BCIs have been used as switches to initiate external robotic supports for the rehabilitation exercise.

The proposed online MI-BCI could identify multi-force loads motor imagery, which may have the potential to provide quantitative relationship between the BCI patterns and the imagined force intensities not just as switch. It could be used not only to ensure subjects’ engagement in high effort levels, but also to design a better proprioceptive feedback control. As a result, it could potentially lead to a better rehabilitation effect. Further investigation is required to reach a reliable conclusion in the future.

## Conclusions

In this study, we used an online experiment to confirm the feasibility of an MI-BCI based on multiple force loads on a single limb. With a higher force load motor imagery, a higher ERD appears both in mu and beta rhythms. The proposed paradigm in this study provides a method for extending the size of the MI-BCI command set, which may have the potential to design a better therapy of post stroke patients during rehabilitation therapy.

## Abbreviations

BCI: Brain-computer interface
EEG: Electroencephalogram
EMG: Electromyogram
ERD: Event-related desynchronization
ERS: Event-related synchronization
ERSP: Event-related spectral perturbation
fMRI: Functional magnetic resonance imaging
MEH: Motor execution high
MEL: Motor execution low
MI-BCI: Motor imagery based BCI
MIH: Motor imagery high
MIL: Motor imagery low
Multi-CSP: Multi-Common spatial pattern
MVC: Maximum voluntary contraction
RX: Relaxed
SVM: Support vector machine
